# Fairness of the Distribution of Public Medical and Health Resources

**DOI:** 10.3389/fpubh.2021.768728

**Published:** 2021-11-10

**Authors:** Lida Pu

**Affiliations:** ^1^School of Public Administration, Central South University, Changsha, China; ^2^School of Business, Hunan University of Technology, Zhuzhou, China

**Keywords:** public healthcare, health resources, health resource allocation, resource justice, physical health

## Abstract

The fairness of health services is an important indicator of the World Health Organization's performance evaluation of health services, and the fairness of health resource allocation is the prerequisite for the fairness of health services. The research in this article aims to explore how to use health and medical resources fairly and effectively to allocate health resources in different fields, populations and projects, in order to achieve the maximization of social and economic benefits of health and medical resources. In the study of the distribution and equity of public health and medical resources, we comprehensively apply Gini coefficient, Theil index, Lorentz curve and difference index, based on the theory of health resource allocation and the theory of health equity, the province's health service resources have been researched and evaluated, combined with regional health planning theories and public health theories, a variety of scientific methods were used to analyze community health service resources at all levels across the country. At the same time, we reviewed the journal literature about the treatment of patients and children, and analyzed the patients admitted to medical institutions in various regions. The research in this paper found that from 2016 to 2020, the Gini coefficient of the province's health institutions according to population distribution has been fluctuating between 0.14 and 0.17. During this 5-year period, the Gini coefficient of the distribution of medical and health expenditures by population shows a downward trend year by year. From 2019, reach below 0.1, this shows that the fairness of the allocation of health resources according to population has a clear trend of improvement.

## Introduction

Since the reform and opening up, our country's health care has developed rapidly, and the health of the people has been greatly improved. With China's accession to the WTO, the primary problem facing the Chinese government and the medical and health system is how to adapt to the rapid changes in domestic and foreign events and formulate a consistent health policy. The 17th National Congress of the Communist Party of China proposed to build a well-off society in an all-round way, and to create a new situation of socialism with Chinese characteristics as its work goal. At the same time, it calls on the medical and health industry to “create and improve the medical and health system and health level, and promote the physical and mental health of the people” as an important part of improving people's lives and promoting healthy development. In addition, the new round of medical reform proposes that medical and health services always maintain a public welfare nature. In order to achieve the goal of everyone enjoying basic medical and health services, this stipulates that the government's regulation of health resource management becomes the main choice, and the government's macro-control must be led to achieve fairness and effectiveness. At the same time, the people actively cooperate with the government's actions, raise problems and solve them with the joint efforts of the government and the people, so as to make the distribution of public health resources fair and stable.

Physical health is the basis for people to engage in other activities. Medical and health activities are social activities that maintain and restore people's basic survival and activity skills. The prerequisite for the smooth progress of medical and health activities is the reasonable management of medical resources and health resources, and reasonable enjoyment of medical and health resources is the fundamental guarantee for safeguarding the interests of human health. It is a key content related to the overall development of the health industry and a necessary condition for the stable, coordinated and healthy development of the pharmaceutical and health industry. How to manage health resources scientifically and rationally, make full use of limited resources, better serve the people, and improve people's physical and mental health has become an urgent problem to be solved.

Combined with the research progress at home and abroad, different scholars have different views and explorations on such issues. Sam and Nunn criticized the current knowledge about patient and public participation, here called patient and public participation (PPI), and called for the development of robust and theoretically based strategies throughout the continuity of medical education. The study draws on a series of relevant literature and regards PPI as a response process related to the patient-centered learning agenda (1). Cheng et al. proposed an attention-based two-way gated recurrent unit (AB-GRU) medical migration prediction model to predict which hospitals patients will go to in the future (2). The focus of Embrett and Randall's research is to understand how the framework of the problem affects government decisions related to the cancellation of medical service funding. To achieve this goal, a framework describing how the problem frame or explanatory narrative affects government policy decisions was developed and applied to actual cases (3). Osadchuk et al. research aims to ensure the protection and enhancement of everyone's physical and mental health by providing high-quality medical and preventive care, and to maintain their long-term active life. Although human health does not have an accurate market price, it has the highest value for society and individuals (4). Mohammad et al. study investigated the prevalence and related risk factors of job burnout among public service medical staff in Kota Kinabalu, Sabah who participated in the fight against the Covid-19 epidemic. A cross-sectional study was conducted involving 201 medical personnel working in all government hospitals and health clinics (5). Ruiz-Mejía and Méndez-Durán studied the reasons for the high incidence of chronic non-communicable diseases such as diabetes, systemic hypertension, overweight, obesity, dyslipidemia and metabolic syndrome (6). Feng and Pan's research on telemedicine allows limited available medical resources to be shared and fully utilized, and many economically underdeveloped provinces can enjoy higher-level medical sharing services. The overall design of the public health emergency management system will be based on the Internet of Things in accordance with system functions and low latency (7). In order to capture the impact of the spatial heterogeneity of the resources available in the environment and the public health system on the persistence and extinction of infectious diseases, Jlng GE proposed a simplified spatial SIS reaction diffusion model. This model has the allocation and utilization efficiency of medical resources (8). Although these studies give the impact of medical services on expenditures, they are only one-sided and do not analyze the fairness of the allocation of public medical and health resources in terms of population distribution and geographic location.

The innovations of this article are embodied in several aspects: first, it essentially explains the importance of fair distribution of public resources, and puts forward the principles and standards of fair distribution. Second, analyze whether residents have equal access to public resources from the perspective of knowledge and geographical differences. Third, in terms of analysis methods, the method of combining statistics and qualitative analysis is used to analyze the effectiveness of health resource allocation and public health benefits. At the same time, through field research and empirical analysis, the factors that affect the effective allocation of public health resources are explored.

## Methods on the Fairness of the Distribution of Public Medical and Health Resources

### Allocation of Health Resources

Health resource allocation refers to the distribution and transfer (flow) of health resources to health departments and health care providers. It is the government or the market that enables health resources to be appropriately allocated to different types, different departments, different institutions, different projects, and different groups of people, thereby increasing the benefits of the community and the value of health resources.

**Figure 1:**
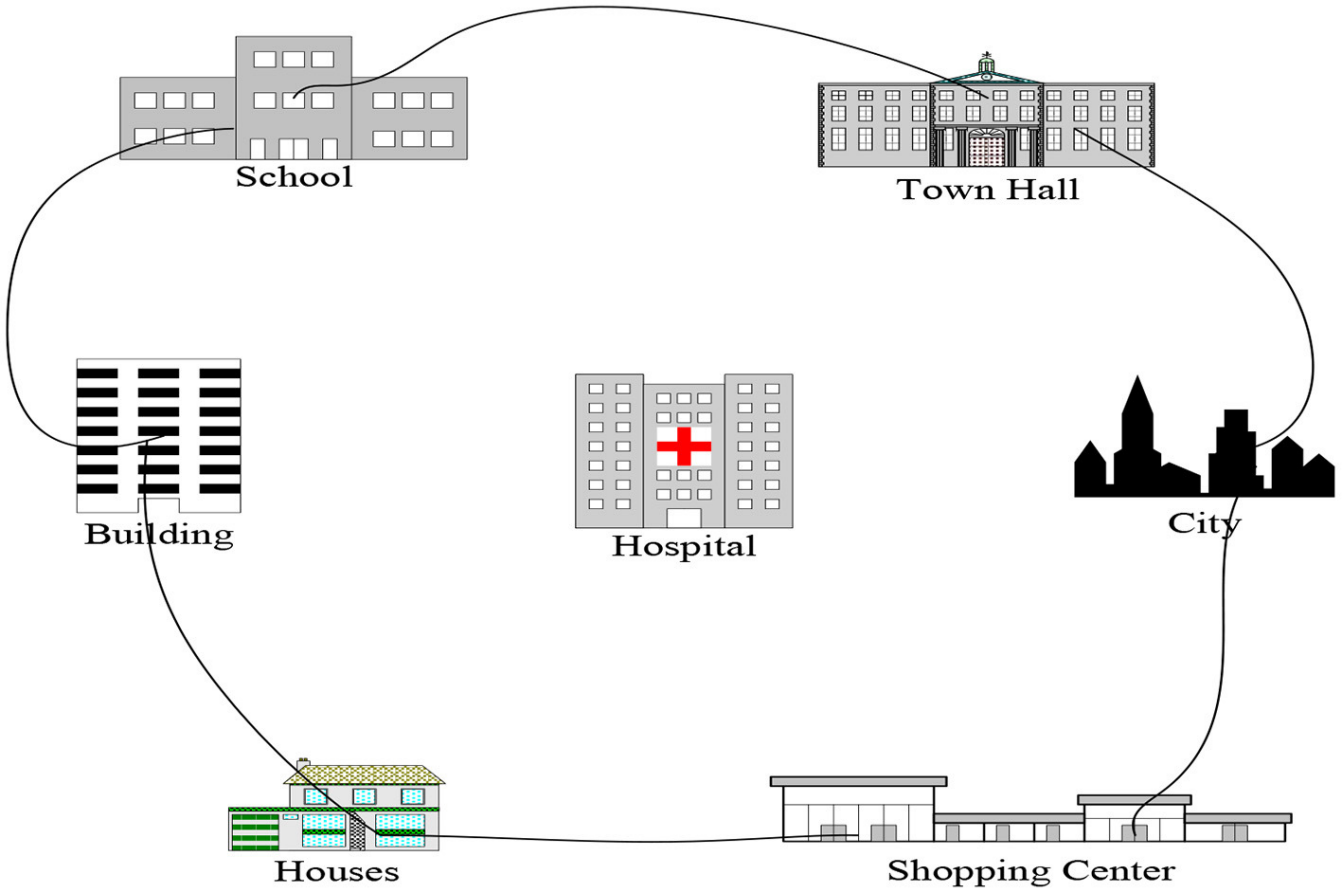
Allocation of health resources.

**Figure 2:**
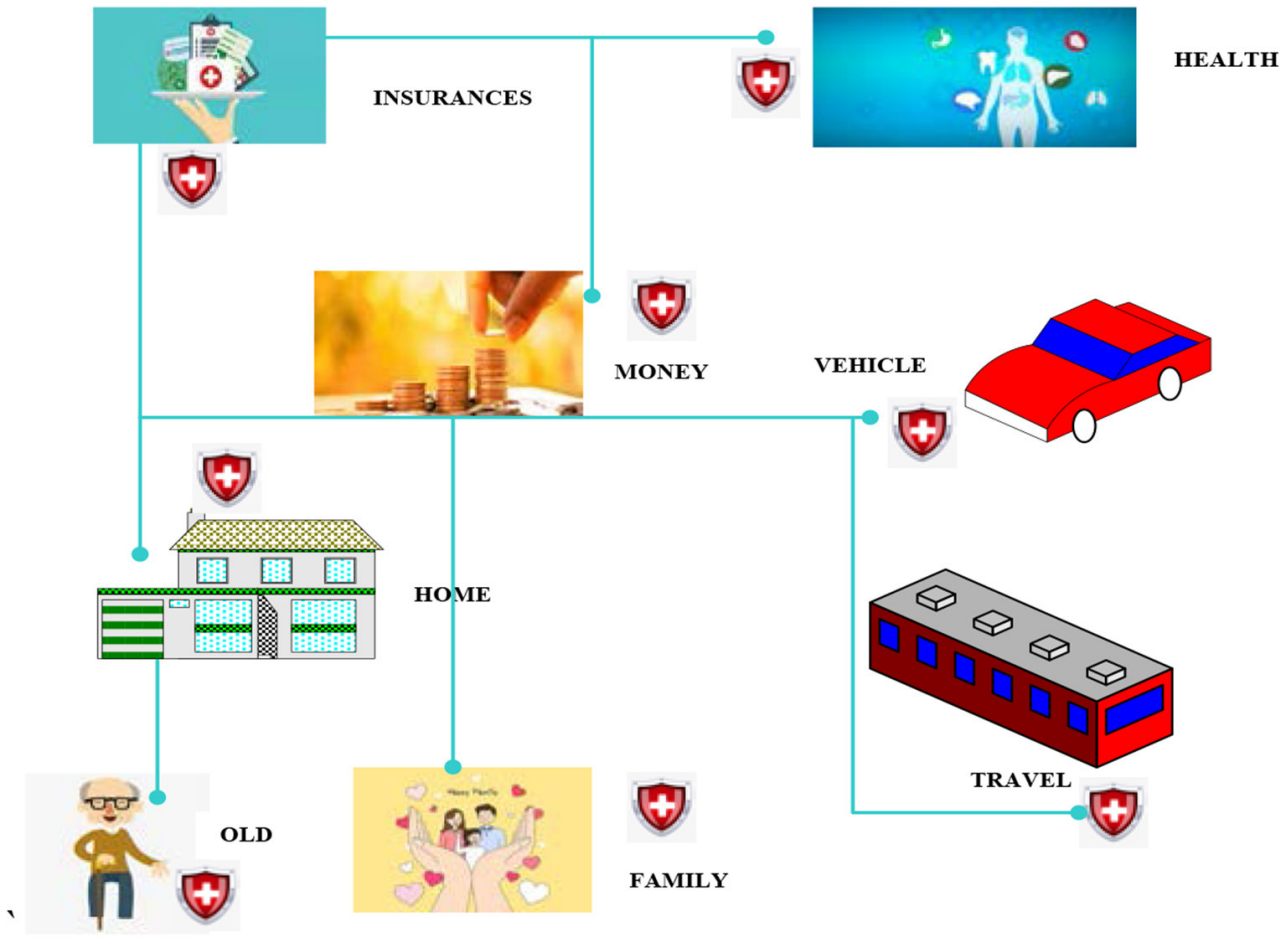
Health care and life.

It can be seen from Figure 1 that all social resources are inseparable from health and medical resources. At the same time, in Figure 2 we can also see that health care is closely related to our lives. The development of health services has become an important indicator to measure the comprehensive strength of a country and region. How health resources are allocated determines whether health output can meet the goals of comprehensive social and economic development with high quality and quantity. This is related to the basic living issues of hundreds of millions of people. It is related to the development level of the whole country's health service (9). Today's medical and health services have undergone major changes compared with those before the reform and opening up. Today's medical and health services can guarantee people's basic living needs and can make people sick and treatable. Before the reform and opening up, our country realized the allocation of health resources through a planning mechanism. The main problem with this allocation model is that it cannot meet the requirements of multi-level growth and large-scale production of medical care in rural and urban communities. Under the condition of limited government finances, the overall allocation of health resources is insufficient and the allocation efficiency is relatively low; with the transition of the financial system from a planned econoour to a market econoour, the government's control over medical institutions has gradually loosened, and the national medical reform has also moved toward a market-oriented path, advocating market-oriented configuration and operation. The proportion of government investment in the medical and health field has fallen, and medical expenses have risen rapidly. Under this model, although it is possible to rapidly increase health resources and improve allocation efficiency to a certain extent, due to the profit-seeking characteristics of the market, the end result is that health resources flow to areas with more developed economic development and strong ability to pay, while ignoring the fairness of health resource allocation, leading to the lack of public welfare in public hospitals. There has been a serious problem of “difficult and expensive medical treatment,” which eventually led to a decline in the efficiency of medical and health services, which obviously deviated from the Pareto efficiency curve. Therefore, excessive commercialization has not only brought social injustice, but also caused the loss of profitability (10). Therefore, in order to ensure fairness, the government must actively and reasonably participate in the allocation of health resources, and at the same time, it should give full play to the role of the market mechanism, improve the efficiency of health resource allocation. It is hoped that in the future, the fairness of the distribution of public medical and health resources will be realized, the problems of “difficult and expensive medical treatment” will no longer arise, and the allocation of health resources will eventually be shared by all.

### Principles of Health Resource Allocation

#### Principles Based on Health Needs and Medical and Health Service Needs

The objects of medical and health services and the end of the service process are people in society. Therefore, the allocation of medical and health resources should reflect the people-oriented concept, focusing on ensuring the health of the whole people, and improving people's health and quality of life. The needs of society determine the provision of medical and health services, and the allocation of medical and health resources should be oriented to meet social needs. Coordinating and rationally distributing limited medical resources and health resources through medical and health service design, and effectively balancing regional surplus or shortage of medical and health resources through resource allocation and redistribution (11).

#### The Principle of Adapting to Economic and Social Development

Reform and opening up and economic globalization have promoted social and economic development, and extensive social changes have also followed. The improvement of people's living standards and the increase in health awareness put higher demands on our country's medical and health services. It is bound to affect and change the allocation of medical and health resources in terms of total, quality, and structure (12). The increasing purchasing power and demand of residents for medical and health services makes the provision of medical and health services not only characterized by sufficiency and diversity, but also taking into account the important factors of the quality, balance and sustainability of medical and health services, to adapt to economic and social development.

#### The Principle of Fairness and Efficiency

Fairness and effectiveness is an important principle for the government to allocate medical and health resources, and it is also a long-term goal (13). Equity in the field of health services refers to ensuring that every member of society has an equal opportunity to enjoy medical and health services based on the allocation of medical and health resources on demand. Medical and health efficiency includes two concepts of econoour and ethics, that is, unreasonable allocation of health resources is both a waste and immoral (14). Paying attention to justice and fairness can not only promote the realization of fairness and fairness and the improvement of human health, but also an important guarantee for health and sustainability, and the sustainable development of health services.

#### Ensure the Principle of Focusing on the Overall Situation

The requirement of “prevention first, grassroots as the focus, and equal emphasis on Chinese and Western medicine” is a policy guideline for improving the status quo of medical and health care. Due to the relatively slow development of the rural econoour and poor basic medical and health conditions, the current situation of rural medical and health is far from that of cities. The degree of improvement in rural medical and health conditions is related to the reform process and development trend of our country's overall medical and health services (15). The allocation of medical and health resources must start from the grassroots level, focusing on improving the level of rural medical and health care. Medical and health investment and related policies need to focus on rural areas and strive to narrow the gap between urban and rural areas. Implement basic medical and health services in rural areas, and ensure that farmers enjoy fair and effective rights to basic medical and health care. Effectively solve the major medical and health problems in rural areas such as difficulty in seeing a doctor, expensive medical care, poverty due to illness, and illness due to poverty, etc., it is necessary not only to improve the health of rural residents, but also to achieve social fairness and justice in the process of medical and health resource allocation (16).

### Commonly Used Methods of Research on Equity of Health Resource Allocation

#### Gini Coefficient

The Gini Coefficient (Gini) is a widely used analysis indicator to measure the income gap of residents in a country (or region). Calculate the Gini coefficient according to the Lorentz curve and the type of Gini coefficient. Quantitative indicators are obtained through data processing and figure area estimation, and the degree of difference in the allocation of medical and health resources is analyzed (17). When the Gini coefficient is lower than 0.2, it means that the allocation of medical and health resources is absolutely even; 0.2 to 0.3 means that the configuration is relatively average, and there is a small gap;0.3 to 0.4 means that the configuration is relatively average and reasonable, but there is a certain gap; 0.4 to 0.5 means that the configuration gap is large. Above 0.6 indicates a huge gap in allocation, and 0.4 is used as a warning line for the gap in resource allocation. As follows:


(1) $$\begin{array}{l} G=\overset{n}{\underset{i=1}{\sum }}A_{i}B_{i}+2\overset{n-1}{\underset{i=1}{\sum }}A_{i}\left(1-C_{i}\right)-1 \end{array}$$

Among them, *A*_*i*_ is the ratio of the population (or geographic area) of each region to the total population (or total geographic area); *B*_*i*_ is the ratio of the number of health resource indicators in each region to the total number of corresponding health resource indicators; *C*_*i*_ = *B*_1_ + *B*_1_ + …*B*_*i*_ is the cumulative percentage of health resources. The Gini coefficient is between 0 and 1. The closer the Gini coefficient is to 0, the fairer the distribution of medical and health resources; the closer the Gini coefficient is to 1, the more unfair the medical and health resources are (18).

#### Theil Index

The Theil index is an important indicator to measure the balance of social resource distribution in a region. In Western science, the Theil index is a method of measuring balance. It checks the fairness and inequality of the distribution of resources by checking whether the weight of the population corresponds to the weight of its income (19). The Theil index value is >0, and its value is smaller, which indicates that health resources can be allocated in this field more effectively. The calculation formula of Theil index is as follows:


(2) $$\begin{array}{l} T=\overset{n}{\underset{i=1}{\sum }}A_{i}\log\left(\frac{A_{i}}{B_{i}}\right) \end{array}$$

In the formula, if *A*_*i*_ is the proportion of each city's population in the province's total population; then *B*_*i*_ is the proportion of each city's health resources in the province's total health resources.

Decomposition of Theil index:


(3) $$\begin{array}{l} T_{\text{total}}=T_{\text{between groups}}+T_{s} \end{array}$$

(4) $$\begin{array}{l} T_{s}=\overset{w}{\underset{j=1}{\sum }}A_{j}T_{j} \end{array}$$

(5) $$\begin{array}{l} T_{\text{Between groups}}=\overset{m}{\underset{j=1}{\sum }}A_{\text{j}}\log\frac{A_{j}}{B_{j}} \end{array}$$

In the formula, *T* is always the overall difference; within the *T* group is the difference in the allocation of health resources in Type I, Type II, and Type III areas. Among the *T* groups are the differences in the allocation of health resources among the Type I, Type II, and Type III areas. *A*_*j*_ is the ratio of the population of each region to the total population. *B*_*j*_ is the ratio of the amount of health resources in each region to the total amount of health resources. *T*_*j*_ is the Theil index of each region. Contribution rate of differences within and between groups to the total Theil index: contribution rate of difference within groups = within T group/T total; contribution rate of difference between groups = T between groups/T total.

#### Lorentz Curve

American economic statistician M. Lorenz first proposed the Lorenz curve to study the optimal allocation of urban and rural medical and health resources, and to study the rational distribution of property, land and income (20).

Suppose the distribution density function of the income variable a is *s(a)* (that is, the percentage of the population with income *a* to the total population), the total population is *M*, then the population whose income is <*g* is $\int_{0}^{g}Ms\left(a\right)da$. Its percentage of the total population is:


(6) $$\begin{array}{l} S\left(g\right)=\int_{0}^{g}Ms\left(a\right)da/M=\int_{0}^{g}s\left(a\right)da \end{array}$$

The sum of the income of all people whose income is <*t* (called cumulative income) is $\int_{0}^{g}aMs\left(a\right)da$. Its proportion in total income is:


(7) $$\begin{array}{l} H\left(g\right)=\int_{0}^{g}aMs\left(a\right)da/\int_{0}^{\infty }aMs\left(a\right)da=1/a\int_{0}^{g}as\left(a\right)da \end{array}$$

Among them is the expected value of income $a=\int_{0}^{\infty }as\left(a\right)da$ or the total average income of the society. By the parametric equation:


(8) $$\begin{array}{l} S=S\left(g\right)=\int_{0}^{g}S\left(a\right)da\left(g\geq 0\right) \end{array}$$

(9) $$\begin{array}{l} H=H\left(g\right)=1/a\int_{0}^{g}as\left(a\right)da\left(g\geq 0\right) \end{array}$$

**Figure 3:**
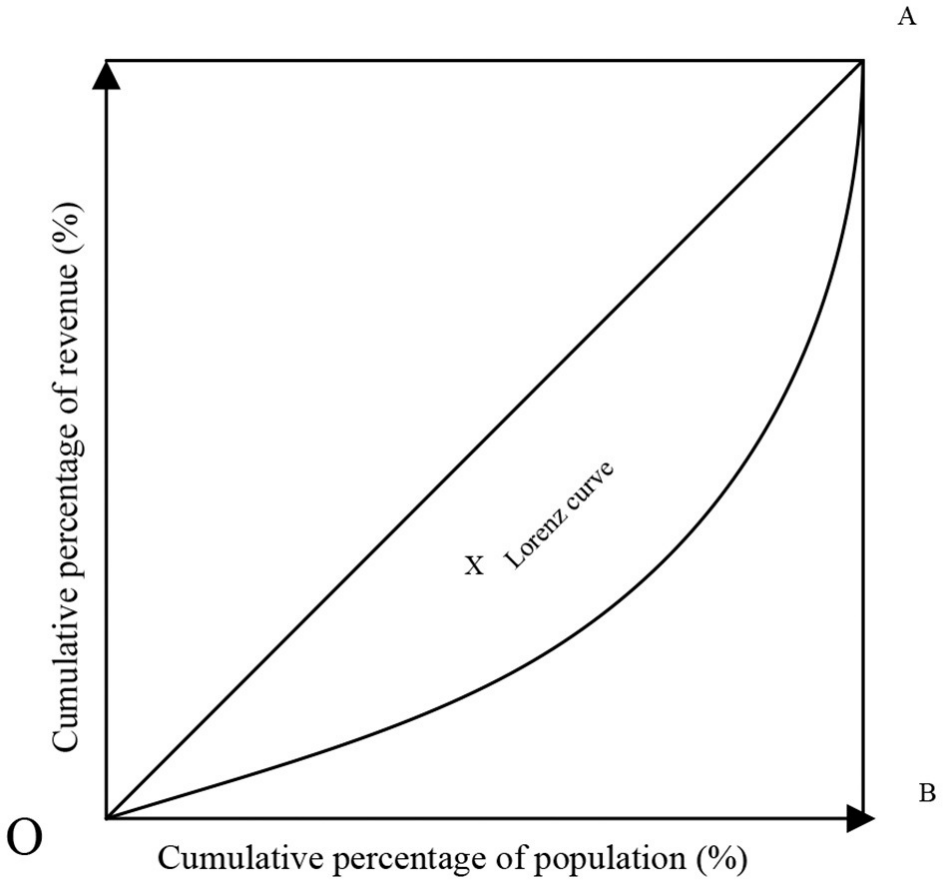
Lorentz curve.

Figure 3 the horizontal axis of the Lorenz curve is the cumulative percentage of the population, and the vertical axis is the corresponding cumulative percentage of medical resources. If the curve coincides with the diagonal, it indicates that the health resources are at an absolute average level. If the curve is close to the diagonal, it means that social health resources are loosely distributed and low-income groups appear; if the curve is close to the diagonal, it means that it is below the diagonal and inclined to the horizontal axis. It can be seen that the fairness of social health resources is returning, mainly in high-income groups.

The Lorentz curve has the following properties:

(1) S(0) = 0, H(0) = 0, that is, 0% of the population's income accounts for 0% of the total income; and S(∞) = 1, H(∞) = 1, that is, 100% of the population revenue accounts for 100% of total revenue.

(2) The Lorentz curve is increasing because:


(10) $$\begin{array}{l} dH/dS=dH/dS÷dS/dg=gs\left(g\right)/as\left(g\right)=g/a\geq 0 \end{array}$$

(3) The Lorentz curve is convex downward because:


(11) $$\begin{array}{l} D^{2}H/dS^{2}=d\left(dH/dS\right)/dS\\ \;=d\left(g/a\right)/dg÷dS/dg=1/as\left(g\right)\geq 0 \end{array}$$

#### Dissimilarity Index

The dissimilarity index (ID) is to use the health proportion of a certain class and the relative population proportion of this class to analyze the concentration of health in a certain class or evenly distribute in each class, and evaluate the degree of health difference of each class (21, 22). If there are *N* = 1, 2, 3,., n social classes, the dissimilarity index is:


(12) $$\begin{array}{l} ID=\frac{1}{2}\overset{i}{\underset{n=1}{\sum }}|T_{nx}-T_{ny}| \end{array}$$

Among them, Tnx is the proportion of the population in the Nth class, and Tny is the proportion of the population in the Nth class. The greater the difference between the two, it shows that the higher the degree of unfairness in the health of this class. The ID value is between 0 and 1. The smaller the ID value, the more uniform the health distribution, and the larger the ID value, the more uneven the health distribution.

#### Concentration Index Method

The concentration index method (CI) is a more appropriate method to measure the degree of health inequality related to socio-economic conditions. This is an index recommended by the World Bank to assess the inequality of health services under different socio-economic conditions (23, 24). The concentration index is sorted according to the per capita net income level, so that the research variable is related to the personal economic income, which can well-reflect the impact of the economic level on the equity of the allocation of health resources. It is a method to assess the degree of per capita health inequality and measure the degree of health inequality related to socio-economic conditions (25).

The calculation formula of the concentration index is:


(13) $$\begin{array}{l} M=\frac{1}{2}\overset{n-1}{\underset{i=0}{\sum }}\left(A_{i}+B_{i+1}\right)\left(A_{i+1}-A_{i}\right) \end{array}$$

(14) $$\begin{array}{l} A_{0}=0,B_{0}=0 \end{array}$$

(15) $$\begin{array}{l} CI=2\times \left(0.5-S\right) \end{array}$$

In the formula, the value of A is arranged according to the annual per capita disposable income of each resident from small to large, and then the cumulative percentage of the population in each region is calculated; B value is the cumulative percentage of community health service resource allocation; i is the ranking of each region in descending order of economic level, and n is the number of regions. The value range of the concentration index CI is [−1, 1], when its value is 0, it means fairness or equality, when its value is positive, it means that the allocation of health resources is concentrated in higher-income groups, and there is inequality in favor of the rich; when its value is negative, it means that the allocation of health resources is concentrated in lower-income groups, and there is inequality in favor of the poor.

#### DEA Measurement Algorithm

The DEA calculation method is used to measure the efficiency of medical and health resources in various provinces. Under the established conditions, the investment of financial resources has achieved the maximum output of medical and health services, then the efficiency of the medical and health expenditure of the place is effective. That is, it is efficient to transform financial resource input into medical and health service output. The calculation method can be expressed by the following linear programming equation:


(16) $$\begin{array}{l} Maximize\;\frac{\overset{k}{\underset{a=1}{\sum }}m_{a}t_{ao}}{\overset{w}{\underset{b=1}{\sum }}h_{b}k_{bo}}=\theta \end{array}$$

(17) $$\begin{array}{l} Subject\;to\;\frac{\overset{k}{\underset{a=1}{\sum }}m_{a}t_{aj}}{\overset{w}{\underset{b=1}{\sum }}h_{b}k_{bj}}\leq 1\left(j=1,2\cdots n\;m_{a}\geq 0,h_{b}\geq 0\right)\; \end{array}$$

#### Evaluation Method of Resource Utilization Efficiency

The rank sum ratio method (RSR) is a non-parametric evaluation method proposed by Chinese statistician Tian Fengtiao. It has the advantages of overcoming the shortcomings of individual indicator analysis and reflecting the comprehensive level of multiple evaluation indicators, and is widely used in the field of health management. Formula for calculating rank:


(18) $$\begin{array}{l} RSR=\sum_{a}^{R}a\times b \end{array}$$

In the formula, *a* is the number of indicators, and b is the number of groups.

#### Calculate the Weighted Rank Sum Ratio (WRSR) of Medical and Health Resource Allocation

The weighted WRSR value calculation formula can calculate the weighted rank sum ratio of medical and health resource allocation:


(19) $$\begin{array}{l} WRSR=\frac{1}{k}\overset{a}{\underset{b=1}{\sum }}X_{b}Y_{cb} \end{array}$$

where *k* is the sample content, and *a* is the number of evaluation indicators. Using the probability unit PRobit as the independent variable and WRSR as the dependent variable, calculate the linear regression equation, namely:


(20) $$\begin{array}{l} WRSR=-0.8546+0.2699PRobit \end{array}$$

## Experiment on the Fairness of Public Medical and Health Resource Distribution

There are many methods to study the fairness of community health service resource allocation, but any method has certain limitations or deviations and cannot fully reflect the fairness of community health service resource allocation. The main reason for the deviations in the reform of the medical and health system is that some regions push all local public hospitals to the market indiscriminately, and some places propose to transplant the property rights reform of industrial and commercial enterprises and sell public hospitals; In addition, some hospitals that are obviously public welfare have also adopted certain market-oriented practices, which aroused people's discussion and dissatisfaction. Therefore, the basic starting point of this study is to use multiple methods to evaluate the fairness of community health service resource allocation from different angles. At present, the academic circles generally use Gini coefficient and Theil index as the main indicators to evaluate the equity of health service resource allocation. Although the use is simple and the effect is intuitive, it is not comprehensive, and may cover up some internal unfairness. For example, the Gini coefficient is low, and it seems fair on the surface, but there may be a certain group that over-occupys community health service resources, and there is inequality that is beneficial to this group. Therefore, this study uses Gini coefficient, Theil index and concentration index to make up for the flaws in the research method of the equity of community health service resource allocation.

**Figure 4:**
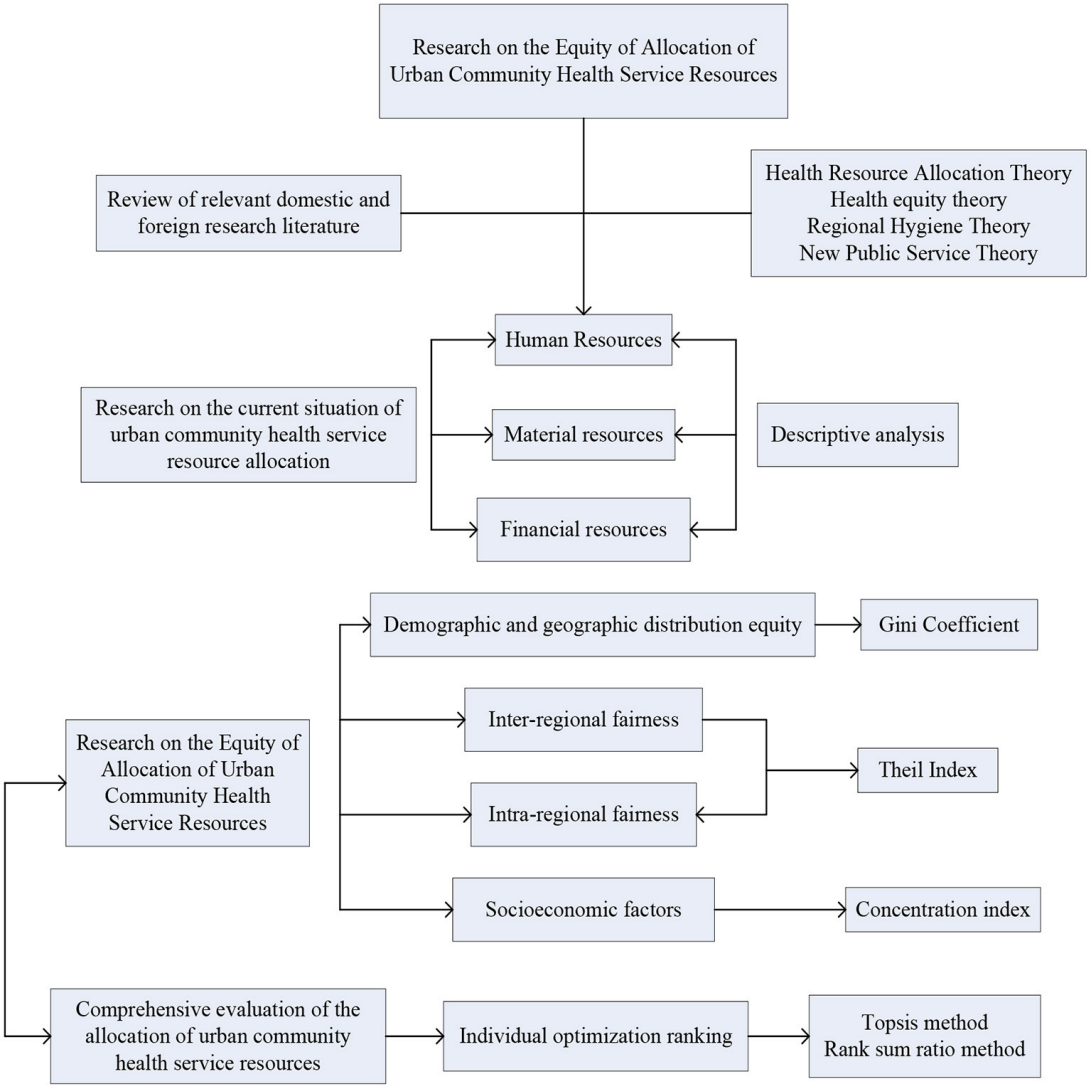
The research framework of the equity of the allocation of urban community health service resources.

Research on the fair distribution of public health resources makes full use of the Gini coefficient, Theil index, Lorentz curve and difference index, based on the theory of health resource allocation and the theory of health equity, the urban resources of community health services are evaluated, and a variety of methods are used to analyze the fair distribution of urban health service resources. Figure 4 is a research framework for the equity of the allocation of urban community health service resources.

After long-term construction and development, the province's medical and health services have continued to grow, and the basic urban and rural medical safety system has basically taken shape, formed a medical and health service system of a certain scale covering urban and rural areas, consisting of hospitals, public health institutions, and primary medical and health institutions. At the end of 2019, medical and health institutions developed moderately. In terms of quantity, there will be 7,003 health institutions of various types in the province in 2020, an increase of 0.89% over 2016; among them, there are 232 hospitals in the province, an increase of 3.4% over 2016; the total number of primary medical and health institutions is 6,037, an increase of 8.67% over 2016; 213 professional public health institutions, an increase of 32.67% over 2016; there were 10 other health institutions, a decrease of 35.71% from 2016.

**Figure 5:**
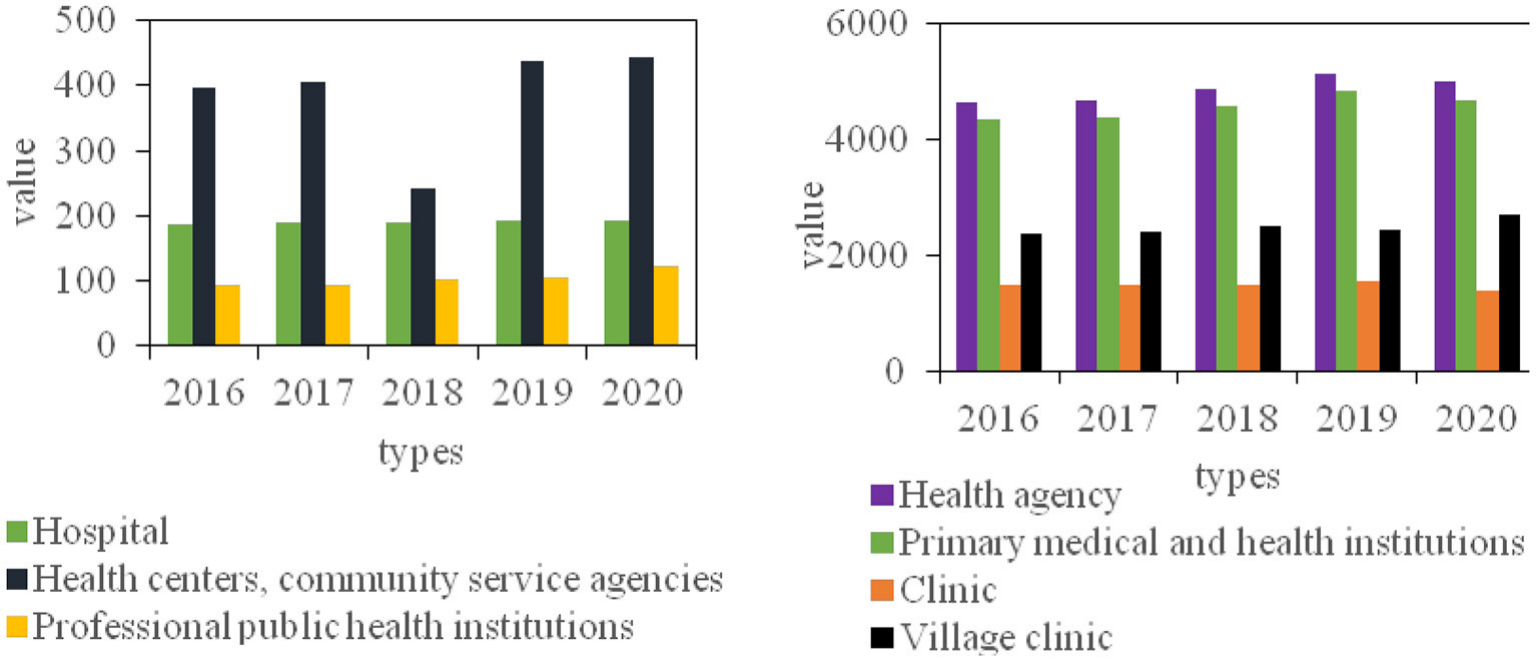
2016-2020 the development trend of medical and health institutions.

It can be seen from Figure 5 that from 2016 to 2020, the number of hospitals has not changed much, and the annual increase or decrease varies between 2 and 3; since 2016, the number of health centers and community service organizations has been on the rise, which is in line with the country's emphasis on improving the three-level medical and health network. Give full play to the leading role of serving prefecture-level hospitals and the backbone role of municipal health centers; from 2016 to 2019, the number of clinics fluctuates slightly, but the overall development is stable, but there has been a large decline in 2020, a decrease of 11.23%; the village clinic has been growing steadily since 2016, but it has declined in 2019, and then saw a substantial increase in 2020, an increase of 12.67% over the previous year.

**Figure 6:**
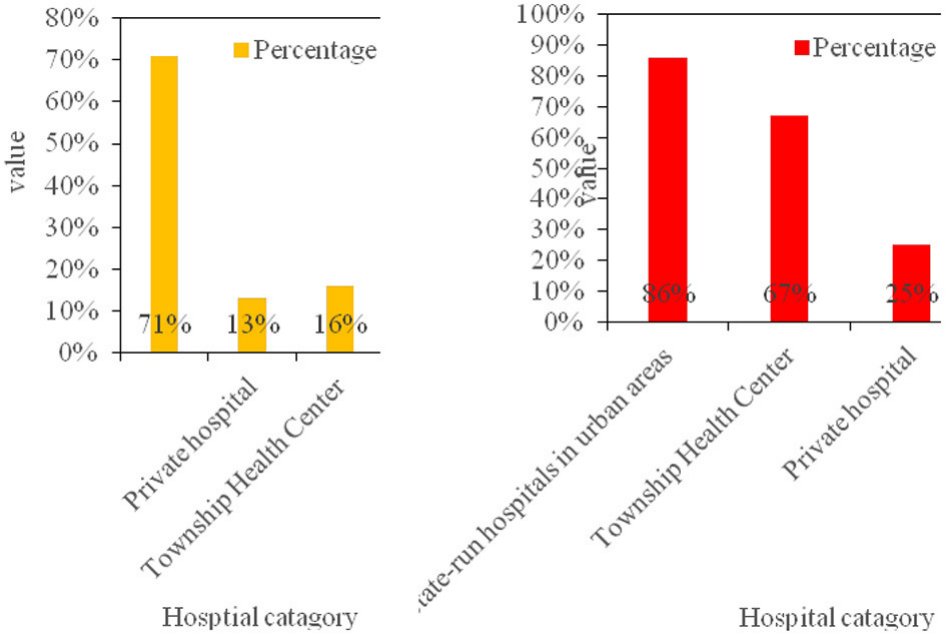
The structure of the number of visits in 2016 and the comparison of the utilization rate of different types of hospitals.

At the same time, the distribution of patients admitted to medical institutions in the region was analyzed. In 2016, the total number of patients receiving treatment was 614,000, and the number of patients treated by health care institutions accounted for 90.2%. The number of diagnosis and treatment in 9 township hospitals accounted for 20.1% of the total number of hospitals under the bureau. As shown in Figure 6.

According to the above data analysis, more people will choose non-profit medical institutions organized by the government, and concentrated in urban hospitals. This is mainly due to economic development, the improvement of people's living standards and scientific lifestyles, the gradual enhancement of human health concepts, and the increasing demand for medical and health services.

## Experimental Data on the Fairness of Public Medical and Health Resource Allocation

### Equity Analysis of Health Resource Allocation Based on Gini Coefficient and Lorentz Curve

**Figure 7:**
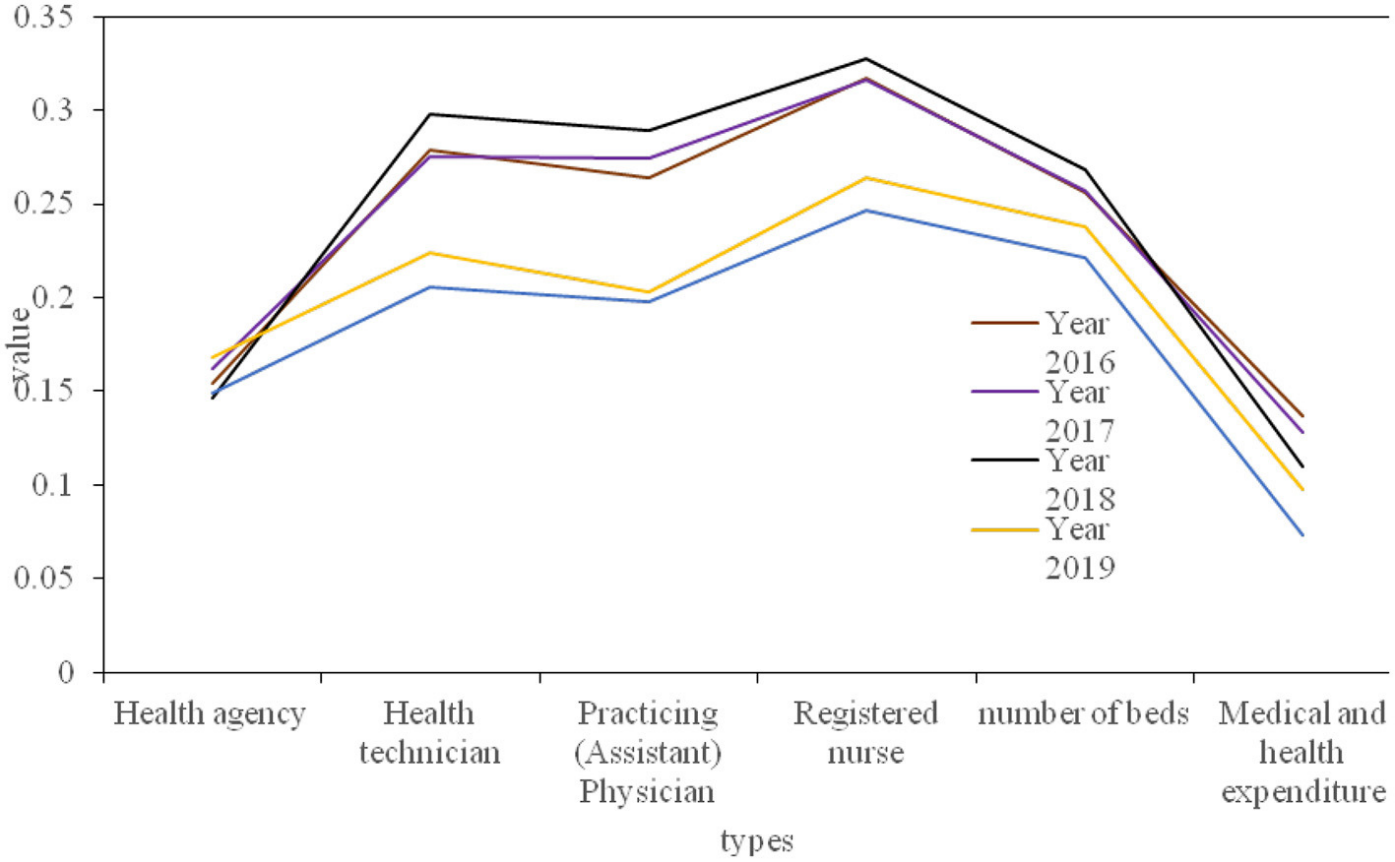
2016–2020 Gini coefficient of the distribution of various health resources by population.

**Figure 8:**
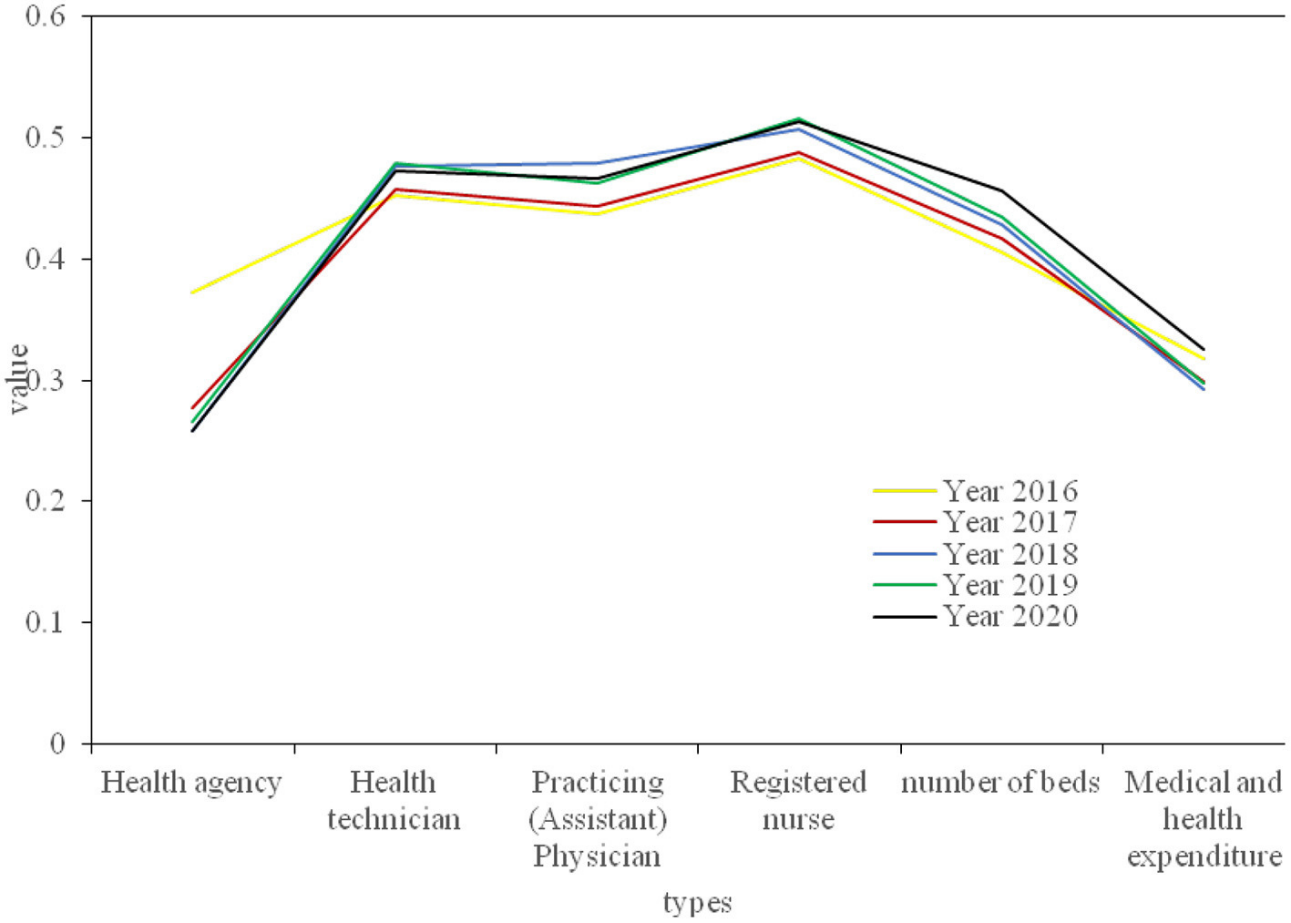
2016–2020 Gini coefficients of various health resources by geographic distribution.

The province's health resources are divided into three aspects: human resources, material resources and financial resources. Human resource allocation data includes the number of health technicians, practicing (assistant) doctors and registered nurses; the material resource allocation data includes the number of health institutions and the number of beds; the financial resources data are mainly medical and health expenditures. Next, calculate the Gini coefficient of each health resource according to the population distribution, and calculate the Gini coefficient of each year according to the geographical area of the province. Figures 7, 8 respectively show the Gini coefficients of various health resources in the province from 2016 to 2020 according to population and geographical distribution. The population distribution is divided according to the number of people in each region, and the geographical distribution is divided according to the different development conditions of each city, which can fully demonstrate whether its resource allocation is fair.

**Figure 9:**
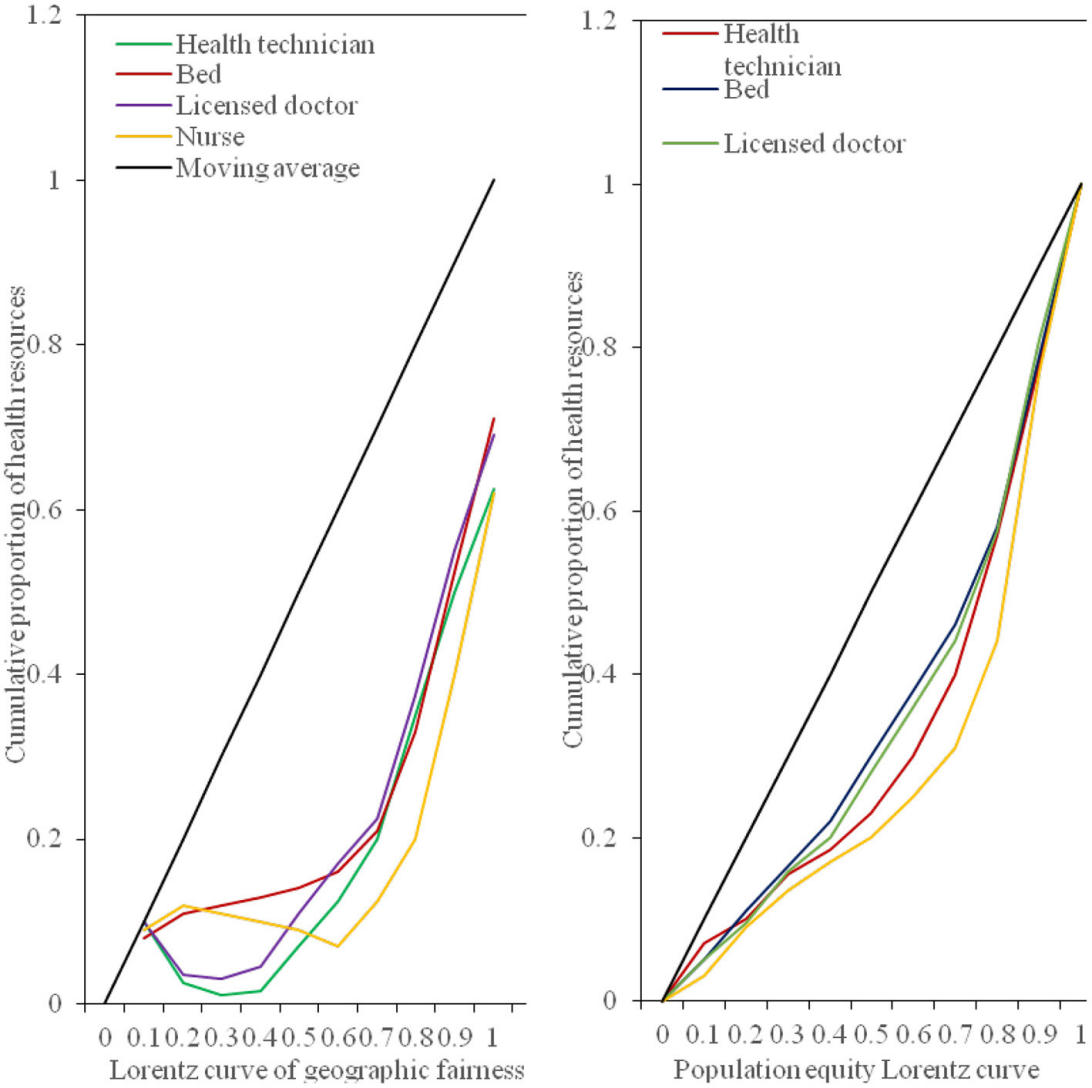
The Lorentz curve of the geographic equity of the medical and health resources population.

At the same time, in order to analyze the fairness of health resources in our province more intuitively, the Lorentz curve is drawn according to the difference of population distribution and geographical location, as shown in Figure 9.

From the perspective of the fairness ranking of various indicators of health resource allocation, regardless of whether it is configured by population or by region, the Gini coefficient of the allocation of health institutions is lower than that of other resource allocations, and its fairness is the best, followed by the distribution of medical and health expenditures. Registered nurses are the least fair indicator of the distribution of health resources. As shown in Table 1. The main reason for the poor fairness of resource allocation for registered nurses is that there are fewer staff engaged in the profession of nurses, which leads to the uneven distribution of nurses.

**Table 1:** The fairness ranking of various indicators of medical and health resource allocation.

	**Gini coefficient by population distribution**	**Gini coefficient by geographical distribution**
Health agency	1	1
Health technician	4	5
Practicing (assistant) physician	3	4
Registered nurse	6	6
Number of beds	5	3
Medical and health expenditure	2	2

From the above data, it can be concluded that from the perspective of population allocation, from 2016 to 2020, the Gini coefficients of the four indicators of health institutions, health technicians, practicing (assistant) physicians, beds, and medical and health expenditures all fluctuate between 0.07 and 0.30. It shows that these 4 health resources are in the best and fair state according to population allocation. The Gini coefficient of registered nurses is between 0.2 and 0.4, which is fairly fair. From the perspective of geographical distribution, the Gini coefficient of the two indicators of health institutions and medical and health expenditure is between 0.2 and 0.4, which is fairly fair. However, the Gini coefficient of health technicians, practicing (assistant) doctors, registered nurses, and geographically distributed hospital beds exceeds 0.4, and their fairness is in a warning state. The results show that the Gini coefficient of medical and health resources distributed by population is lower than that by geographical distribution, indicating that the fairness of medical and health resources distributed by population is better than that by geographical distribution. From 2016 to 2018, the Gini coefficient of beds, health technicians, doctors and nurses divided by population distribution in the province has not changed much, but by 2019, the Gini coefficient has dropped significantly; from 2016 to 2020, the Gini coefficient calculated by the population distribution of health institutions fluctuated between 0.14 and 0.17, with little change; in the past 5 years, the Gini coefficient of medical and health expenditure by population distribution has shown a downward trend year by year. Since 2019, it will fall below 0.1, but the Gini coefficient of the province's health resources divided by geographical distribution has not changed much. However, the Gini coefficient of various indicators divided by geographical distribution will increase slightly in 2020. This shows that the fairness of distribution of health resources according to population distribution has a significant improvement trend, but the fairness of distribution of health resources according to geographical distribution has declined.

### Theil Index Evaluates the Balance of Health Resource Allocation in the Province

We make statistics on the allocation of bed resources in medical and health institutions in this province. According to the per capita GDP from 2015 to 2020, the province's cluster analysis is divided into 4 categories: Class I areas (economically developed areas), Class II areas (medium economically developed areas), Class III areas (economically underdeveloped areas). Tables 2, 3 respectively represent the Theil index of the province and each region, and the Theil index of the province's bed resource allocation and its decomposition.

**Table 2:** 2015–2020 Theil index by region.

**Years**	**Class I area**	**Class II area**	**Class III area**	**Citywide**
2015	0.051	0.041	0.006	0.125
2016	0.075	0.006	0.201	0.265
2017	0.041	0.029	0.002	0.135
2018	0.061	0.021	0.004	0.098
2019	0.051	0.005	0.021	0.092
2020	0.042	0.002	0.022	0.086

**Table 3:** 2015–2020 Theil index of bed resource allocation and its decomposition.

**Years**	**Interregional Theil index**	**Contribution rate (%)**	**Regional Theil index**	**Contribution rate (%)**	**Total difference**
2015	0.025	19.22	0.086	80.78	0.125
2016	0.022	8.91	0.235	91.09	0.265
2017	0.036	29.98	0.071	70.02	0.135
2018	0.022	19.89	0.081	80.02	0.098
2019	0.028	29.78	0.061	70.22	0.092
2020	0.023	26.89	0.062	73.11	0.086

From the above-stated Theil index, it can be concluded that the total Theil index for the allocation of bed resources in medical and health institutions in the province in 2020 is 0.086. The Theil index for Type I areas is 0.042, Type II areas are 0.002, and Type III areas are 0.022. On the whole, the number of II has dropped, from 0.125 to 0.086. Among them, the Theil index of Type I and Type II areas declined, and the Theil Index of Type III areas increased. Regarding the changes in the Theil index between regions and within the region, from 2015 to 2020, the Theil index for the allocation of bed resources in this province will drop from 0.101 to 0.081. Among them, the inter-regional Theil index decreased from 0.024 to 0.022, and the contribution rate of inter-regional differences increased from 23.66% to 27.64%. The regional Theil index dropped from 0.077 to 0.059, and the contribution rate of regional differences dropped from 76.34 to 72.36%. The population fairness of bed resource allocation in this province is better than geographical fairness. Geographic fairness has long exceeded the unfair warning line. The fairness of population allocation by professional public health institutions is the worst, and the fairness of geographical allocation of hospital bed resources is the worst; the unbalanced allocation of beds is mainly due to the uneven distribution in the region; the number of beds in medical and health institutions in the province has developed greatly, but the structure of bed resources is still unreasonable.

As shown in Table 4, during 2016–2020, the Theil index allocated by health institutions changed within the range of 0.015 to 0.02, with little fluctuation; from 2016 to 2018, the Theil index of health technicians, physicians, nurses, and bed allocation all showed an upward trend, but by 2019, its total Theil index began to decline significantly, while the Theil index for government health expenditure allocation from 2016 to 2020 showed a downward trend. It can be seen that the fairness of the allocation of various health resources in the province is improving year by year. Combined with the analysis of the total Theil index from 2016 to 2020, among all types of health resources, the allocation of nurses is the least equitable, and the allocation of health institutions and government health expenditures is relatively well-balanced.

**Table 4:** Theil index of health resource allocation.

**Years**	**Health agency**	**Health technician**	**Practicing (assistant) physician**	**Registered nurse**	**Bed**	**Government health expenditure**
2016	0.016307	0.043208	0.051012	0.069318	0.042118	0.013922
2017	0.017408	0.047082	0.053418	0.069819	0.040221	0.015913
2018	0.014307	0.054079	0.060122	0.073817	0.050223	0.009721
2019	0.018702	0.037209	0.030132	0.050122	0.039823	0.007211
2020	0.013609	0.017189	0.028326	0.041321	0.032019	0.003909

### Difference Index Evaluates the Fairness of the Allocation of Bed Resources in Our Province

**Figure 10:**
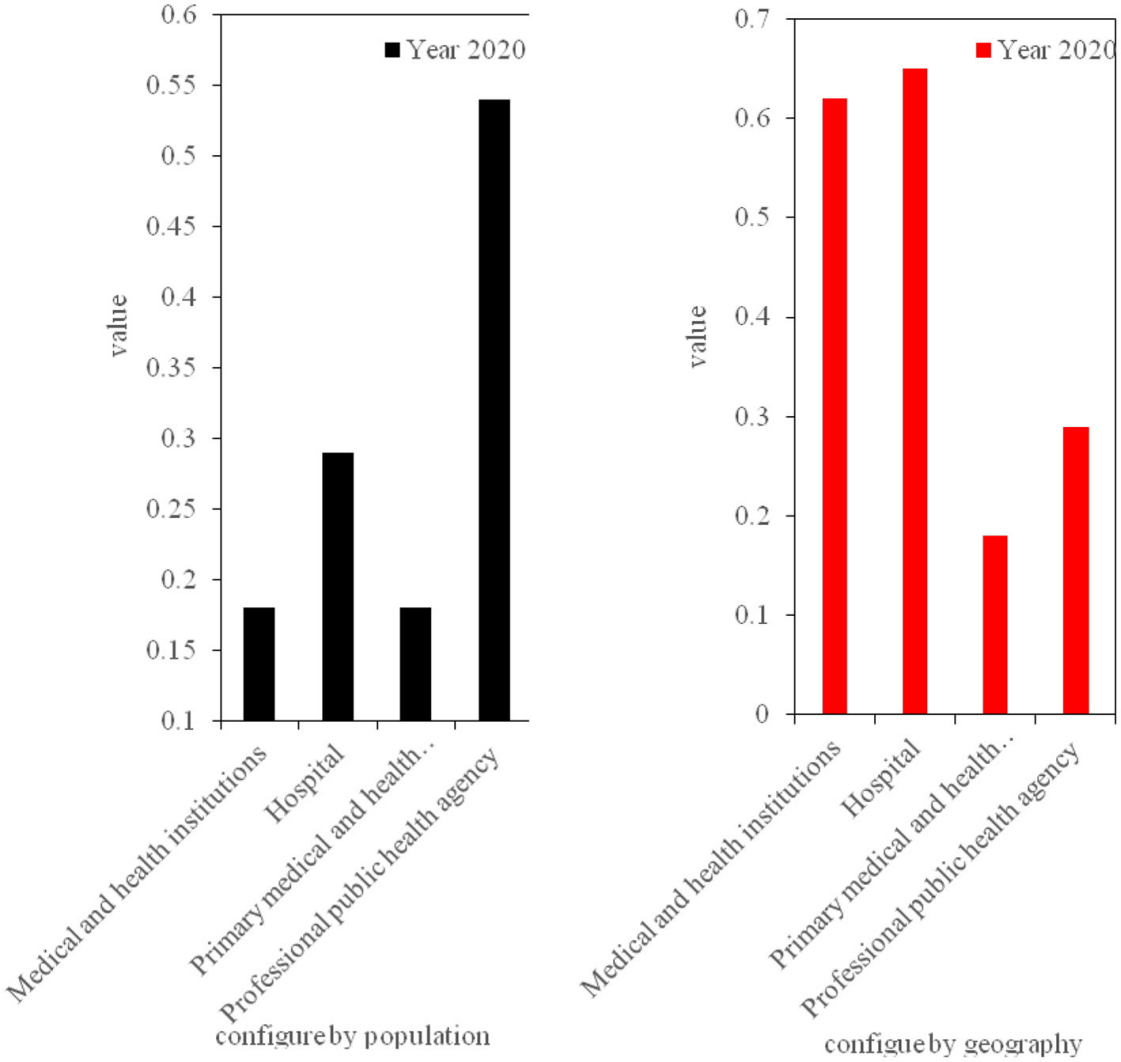
Changes in the difference index of medical institutions.

The difference exponential fairness analysis of the allocation of bed resources in our province in 2020 is shown in Figure 10.

Analyzing the results of the above figure, it can be concluded that in 2020, the difference index of the allocation of beds in medical and health institutions in our province is 0.18 by population and 0.62 by region; among them, according to population allocation, professional public health institutions have the largest difference in bed resource index, which is 0.54. According to the geographical distribution, the hospital bed resource difference index is the largest, which is 0.69. The resource difference index between beds in professional health institutions and hospital beds is relatively large, and its fairness needs to be strengthened.

## Conclusions

This article mainly introduces the relevant theories of medical and health resource allocation. At the same time, it analyzed the allocation of health resources in the province in recent years. Through the Gini coefficient and the Lorentz curve, a statistical analysis of the population distribution and geographic area of our province was carried out, at the same time, the Theil index and the difference index were analyzed for the beds of medical institutions in this province. It was discovered that the province's health resources were unfairly allocated and irrationally allocated in the early stage, but they were improved later. And for this phenomenon, we should also take timely measures, adjust the resource structure, sink high-quality resources, and health administrative departments should explore scientific and reasonable resource allocation policies based on local actual conditions, and update resource allocation standards in a timely manner. Appropriately control the development speed of the hospital bed size, focus on the overall development of medical education and research, and enhance the overall strength; encourage new hospitals to focus on specialized hospitals such as rehabilitation, obstetrics and geriatrics, develop shortage of medical resources, and promote multidisciplinary development; adhere to the combination of prevention and prevention, integrate existing maternity and child health hospitals (institutions), occupational disease prevention and treatment hospitals (institutions) and other institutions, and construct a public health system that promotes and develops in coordination, better play the functions of disease prevention and health care and rehabilitation. In addition, the introduction of high-quality medical resources in the surrounding areas through the establishment of branch hospitals, new hospitals or upgrading the level of regional medical centers, etc. The focus of new high-quality resources tends to be the surrounding counties and cities with weak resources, and various methods such as raising the standard of bed allocation and increasing corresponding resource input to adjust the spatial layout of high-quality medical resources, improve the feasibility of medical services for residents in surrounding districts and counties.

## Data Availability Statement

The original contributions presented in the study are included in the article/supplementary material, further inquiries can be directed to the corresponding author/s.

## Author Contributions

The author confirms being the sole contributor of this work and has approved it for publication.

## Conflict of Interest

The author declares that the research was conducted in the absence of any commercial or financial relationships that could be construed as a potential conflict of interest.

## Publisher's Note

All claims expressed in this article are solely those of the authors and do not necessarily represent those of their affiliated organizations, or those of the publisher, the editors and the reviewers. Any product that may be evaluated in this article, or claim that may be made by its manufacturer, is not guaranteed or endorsed by the publisher.

## References

[1] Sam R Nunn S. Towards a pedagogy for patient and public involvement in medical education. Med Educ. (2016) 50:79–92. 10.1111/medu.12880 26695468

[2] Cheng L Shi Y Zhang K. Medical treatment migration behavior prediction and recommendation based on health insurance data. World Wide Web. (2020) 23:1–20. 10.1007/s11280-020-00781-3

[3] Embrett M Randall GE. Understanding government decisions to de-fund medical services analyzing the impact of problem frames on resource allocation policies. Health Care Anal. (2021) 11:1–21. 10.1007/s10728-020-00426-6 33387163

[4] Osadchuk MA Osadchuk AM Korzhenko NP Trushin MV. Econoour and healthcare: their role in providing medical care and maintaining public health. J Adv Res Law Econ. (2019) 10:864–70. 10.14505//jarle.v10.3(41).22

[5] Mohammad AH Shamsudin SB Ahmad Kamarudin N Ching LM Radzran AS Mohd Deris MH. Occupational burnout among public medical officers during the early stage of Covid-19 pandemic in Kota Kinabalu, Sabah. Malays J Public Health Med. (2021) 21:317–26. 10.37268/mjphm/vol.21/no.1/art.893

[6] Ruiz-Mejía R Méndez-Durán A. Public health problem: chronic kidney disease in Mexico, the urgent need to train medical specialists. Gac Medica de Bilbao. (2018) 115:194–9.

[7] Feng Y Pan Z. Optimization of remote public medical emergency management system with low delay based on internet of things. J Healthc Eng. (2021) 2021:1–10. 10.1155/2021/5570500 33854745 PMC8021466

[8] Jlng GE Lin Z Zhu H. Environmental risks in a diffusive SIS model incorporating use efficiency of the medical resource. Discrete Continuous Dyn Syst Ser B (DCDS-B). (2017) 21:1469–81. 10.3934/dcdsb.2016007

[9] Pérez-Molina JA Álvarez-Martínez MJ Molina I. Medical care for refugees: a question of ethics and public health. Enferm Infecc Microbiol Clin. (2016) 34:79–82. 10.1016/j.eimc.2015.12.007 26811213 PMC7103281

[10] Srivastav AK Ghosh M Bandekar SR. Modeling of COVID-19 with limited public health resources: a comparative study of three most affected countries. Eur Phys J Plus. (2021) 136:1–26. 10.1140/epjp/s13360-021-01333-y 33842186 PMC8020377

[11] Lindegren ML Mccormack L Barnes B Mitchel E Jones S Schaffner W. Assessment of administrative medical claims data for public health surveillance of invasive group a streptococcal necrotizing fasciitis in tennessee. Public Health Rep. (2016) 131:560–5. 10.1177/0033354916662214 27453600 PMC4937117

[12] Allgaier RL Shaafi-Kabiri N Romney CA Wallis LA Joseph Burke J Bhangu J. Use of predictive modeling to plan for special event medical care during mass gathering events. Disaster Med Public Health Prep. (2019) 13:1–6. 10.1017/dmp.2019.1 31169107

[13] Nawata K Matsumoto A Kimura M.. Evaluation of the distribution and factors affecting blood pressure using medical checkup data in Japan. Health. (2017) 09:124–37. 10.4236/health.2017.91009

[14] Tabrizi JS Gholipour K Farahbakhsh M Hasanzadeh A. Managerial barriers and challenges in iran public health system: east Azerbaijan health manager's perspective. J Pak Med Assoc. (2017) 67:409–15. 28303991

[15] Bekemeier B Park S Whitman G. Challenges and lessons learned in promoting adoption of standardized local public health service delivery data through the application of the public health activities and services tracking model. Am Med Inform Assoc JAMIA. (2019) 26:1660–3. 10.1093/jamia/ocz160 31550365 PMC7647159

[16] Prinja S Balasubramanian D Jeet G Verma R Kumar D Bahuguna P. Cost of delivering secondary-level health care services through public sector district hospitals in India. Indian J Med Res. (2017) 146:354–61. 10.4103/ijmr.IJMR_902_15 29355142 PMC5793470

[17] Wu Y Huang Y Lu J. Potential effect of medical insurance on medicare: evidence from China. Iran J Public Health. (2016)45:1247–60. 27957431 PMC5149488

[18] Al-Rabiaah A Temsah MH Al-Eyadhy AA Hasan GM Al-Zamil F Al-Subaie S. Middle east respiratory syndrome-corona virus (MERS-CoV) associated stress among medical students at a university teaching hospital in Saudi Arabia. J Infect Public Health. (2020) 13:687–91. 10.1016/j.jiph.2020.01.005 32001194 PMC7102651

[19] Ying CA Cheng WE Long TA Pufei LI Yan XU Suxia HU. Exploration and consideration of the medical alliance modes. Iran J Public Health. (2018) 47:1160–5. 30186788 PMC6123575

[20] Gopichandran V. Placing the “radar” under the radar: ethics of public health surveillance. Indian J Med Ethics. (2017) 3:1–6. 10.20529/IJME.2017.076 28889090

[21] Imperato PJ Bruno DM Sweeney MM. Ensuring the health, safety and preparedness of us medical students participating in global health electives overseas. J Community Health. (2016) 41:442–50. 10.1007/s10900-016-0169-7 26882901

[22] García-Romero A Escribano Á Tribó JA. The impact of health research on length of stay in Spanish public hospitals. Res Policy. (2017) 46:591–604. 10.1016/j.respol.2017.01.00619841027

[23] Kochurov BI Ivashkina IV Fomina NV Ermakova YI. Urban health approach to the study and development of large cities. Geogr Nat Res. (2020) 41:203–10. 10.1134/S1875372841030014

[24] Atolia M Papageorgiou C Turnovsky SJ. Taxation and public health investment: policy choices and tradeoffs. Macroecon Dyn. (2021) 25:426–61. 10.1017/S1365100519000245

[25] Montoya-Bartheleour AG Lee CD Cundiff DR Smith EB. COVID-19 and the correctional environment: the American prison as a focal point for public health. Am J Prev Med. (2020) 58:888–91. 10.1016/j.amepre.2020.04.001 32387174 PMC7164863

